# Ferulic Acid Improves Functional Recovery after Acute Spinal Cord Injury in Rats by Inducing Hypoxia to Inhibit microRNA-590 and Elevate Vascular Endothelial Growth Factor Expressions

**DOI:** 10.3389/fnmol.2017.00183

**Published:** 2017-06-08

**Authors:** Zhenjie Li, Shengyun Wang, Wenfang Li, Hongbin Yuan

**Affiliations:** ^1^Department of Anesthesiology, Shanghai Changzheng Hospital, The Second Military Medical UniversityShanghai, China; ^2^Department of Emergency and Critical Care Medicine, Shanghai Changzheng Hospital, The Second Military Medical UniversityShanghai, China

**Keywords:** Ferulic acid, spinal cord injury, neural stem cells, hypoxia, neurogenesis

## Abstract

Spinal cord injury (SCI) is the leading cause of paralysis, disability and even death in severe cases, and neural stem cells (NSCs) transplant has been employed for repairing SCI. Ferulic acid (FA) is able to promote neurogenesis in various stem cell therapies. We aimed to investigate the effect of FA on NSC transplant therapy, and the underlying mechanism, in improving functional recovery in SCI rat model. A rat model of SCI was established, which then received transplant of NSCs with or without FA pre-treatment. Functional recovery of the SCI rats was then evaluated, in terms of spinal cord water content, myeloperoxidase activity and behavioral assessments. Effect of FA in inducing hypoxia in NSCs was also assessed, followed by identifying the hypoxic regulated microRNA and the subsequent target gene. Transplant of FA pre-treated NSCs improved functional recovery of SCI rats to a more significant extent than NSCs without FA pre-treatment. The beneficial effects of FA in repairing SCI was mediated by inducing hypoxia in NSCs, which in turn inhibited microRNA-590 to elevate vascular endothelial growth factor expression. Our findings support the clinical potential of FA in improving efficacy of NSC transplant therapy for treatment of SCI.

## Introduction

Spinal cord injury (SCI) is the leading cause of paralysis, disability and even death in severe cases (Wu et al., 2012). The incidence rate of SCI in China has recently increased by ∼120,000 new cases per annual, and reached over 1 million cases (Liu et al., 2012). Despite improved medical care, treating SCI patients is still a serious challenge, with slim chance of full functional recovery. Given the unique properties of neuron progenitor cells or neural stem cells (NSCs) in repairing injuries of the nervous system, NSC transplant therapy holds promising potential for SCI patients. Several animal studies have demonstrated the efficacy of NSC transplant in the treatment of SCI. For example, implanting an NSC scaffold into SCI rats could promote long-term recovery of neurological functions (Teng et al., 2002). Engraftment of human NSCs into immune-deficient mice could attenuate injury of the central nerve system (Cummings et al., 2005). Importantly, in a primate SCI model, transplant of human NSC has also exhibited clinical beneficial effects in repairing the injured spinal cords (Iwanami et al., 2005).

Hypoxia, a condition where oxygen supply is reduced, is reported to regulate growth, differentiation and functions of various tissues, including the nervous system (Semenza, 2012). Hypoxia activates the major transcription factor hypoxic-induced factor-1α (HIF-1α), which binds hypoxia response elements (HREs) in the promoter of target genes and regulates their expressions (Semenza, 1996). Of particular interest to our current study, hypoxia was recently reported to be involved in neurorehabilitation (Gonzalez-Rothi et al., 2015). For instance, acute intermittent hypoxia was shown to enhance recovery of breathing capacity after chronic cervical spinal injury (Navarrete-Opazo et al., 2017), as well as increase expressions of growth and neurotrophic factors in non-respiratory motor neurons (Satriotomo et al., 2016). The clinical efficacy of hypoxia has also been recently suggested by recently reported clinical trials, in which acute intermittent hypoxia could improve physical function and health status in patients with partial SCI (Astorino et al., 2015), and promote motor functions in patients with with chronic SCI (Lynch et al., 2016).

Ferulic acid (FA), a hydroxycinnamic acid found in plants, was found to induce hypoxia and enhance angiogenesis of human umbilical vein endothelial cells, via increasing expressions of HIF-1α and vascular endothelial growth factor (VEGF) (Lin et al., 2010). In addition, an FA-loaded hydrogel was able to promote neovascularization from the host tissues, also by inducing hypoxia (Park and Gerecht, 2014). On the other hand, FA has been reported to specifically enhance neurogenic differention in therapies involving different types of stem cells. For example, FA could induce neurogenesis of bone marrow stromal cells *in vitro* (Wang et al., 2005), as well as *in vivo* in a rat model of focal cerebral ischemia (Zhao et al., 2013). Importantly, FA has also been shown to induce proliferation of neural progenitor cells both *in vitro* and *in vivo* (Yabe et al., 2010).

However, to date there has been no study reporting the effect of FA in functional recovery after SCI. Based on all the above reports, we speculated that FA might be able to exhibit benefical effects in NSC transplant therapy for treatment of SCI, possibly through its hypoxia inducing property. We therefore designed the current study to investigate the hypothesis, as well as potential underlying molecular mechanisms.

## Materials and Methods

### NSC Isolation

Adult NSCs were isolated from the spinal cords of six patients as previously described (Mothe and Tator, 2015). All procedures have been approved by the ethics committee of Shanghai Changzheng Hospital, The Second Military Medical University and were in compliance with the research protocols involving human subjects. The periventricular spinal cord tissues were obtained from organ transplant donors with written informed consent. In brief, tissues were aseptically harvested and minced into pieces followed by digestion with Papain (20 U/ml) containing DNase I, 1 mM L-cysteine and 0.5 mM EDTA. Cell suspensions were washed once and centrifuged through a discontinuous density gradient to remove membrane fragments. The cell pellet at the bottom were resuspended and filtered through cell strainer before plating in pre-coated culture plates in serum-free Neurobasal-A medium (Thermo Fisher Scientific, Waltham, MA, United States) containing 20 ng/ml EGF, 20 ng/ml bFGF, 2 mM L-glutamine, 100 μg/ml penicillin-streptomycin, 2% B27, and 2 μg/ml heparin. The cells were cultured at 37°C with 5% humidified CO_2_, in the presence of vehicle controls or 10 μM FA (Sigma–Aldrich, St. Louis, MO, United States) for 24 h (Lin et al., 2010). Hypoxic conditions were established by treating cells with 100 μM CoCl_2_ for 24 h. After treatments, adherent NSCs were harvested by trypsinization and resuspended in PBS containing 4% fetal bovine serum.

### SCI Rat Model

Male Sprague Dawley rats, aged 6–8 weeks and weighing 200–220 g, were used in the study with approval from the animal research review committee of Shanghai Changzheng Hospital, The Second Military Medical University. All methods were performed in accordance with the recommendations in the Guide for the Care and Use of Laboratory Animals of the National Institutes of Health. In a spinal cord contusion injury model, the rats were anesthetized using 3% isoflurane through inhaling, and subsequently received dorsal laminectomy at the level of the ninth thoracic vertebra (T9) followed by contusion injury using the NYU impactor (Gruner, 1992), in which a 10 g weight impact rod was dropped from a height of 15 cm. The exposed spinal cord was then intrathecally administered through a 30-gauge needle with 4 μl of cultured NSCs (5 × 10^5^ cells/4 μl) at the site of the injury. Skin was closed by sutures and the animals were allowed to recover on a heating pad. Postoperative treatments included saline for rehydration and Baytril to prevent urinary tract infection. Following transplant the rats were orally administered with 100 mg/mL enrofloxacin (Bayer Animal Health) in drinking water. Rats were assigned in the following experimental groups: (1) sham (*n* = 12), rats underwent sham operations; (2) SCI (*n* = 12), rats underwent SCI operations; (3) SCI+NSC (*n* = 12), rats underwent SCI operations and injected with PBS-treated NSCs; (4) SCI+FA-NSC (*n* = 12), rats underwent SCI operations and injected with FA pre-treated NSCs; (5) SCI+FA-NSC-control (*n* = 12), rats underwent SCI operations and injected with FA pre-treated NSC stably expressing control miR; (6) SCI+FA-NSC-miR-590 (*n* = 12), rats underwent SCI operations and injected with FA pre-treated NSC stably expressing miR-590.

### Behavioral Assessments

Extent of SCI was evaluated at indicated days post SCI, using Basso, Beattie and Bresnahan (BBB) scores. The locomotor function of rats was determined based on the BBB scale as described (Buller et al., 2010). In brief, locomotor activity was examined over a period of 4 min by independent observers in a blinded manner.

### Spinal Cord Water Content

Spinal cord edema was assessed by the determination of spinal cord water content, which was calculated as follows: spinal cord water % = (wet weight - dry weight)/wet weight × 100%. The dry weight was measured after the injured spinal cords were dried for 24 h at 110°C using the Havppvel method.

### Myeloperoxidase (MPO) Activity

Spinal cord tissues were harvested and weighed at indicated days post SCI. The tissues were processed by MPO (myeloperoxidase) assay kits (Sigma–Aldrich, St. Louis, MO, United States) for determination of the extent of polymorphonuclear leukocyte (PMN) infiltration. The MPO activity was measured by the rate of change in absorbance at 460 nm and expressed as units of MPO (the quantity of MPO enzyme to degrade 1 μmol of peroxide per minute) per gram of wet tissues.

### MicroRNA (miRNA, miR) Assays

Expression of miR-590 was measured with mature miRNA assay kit (478168_mir; ThermoFisher Scientific, Waltham, MA, United States) according to manufacturer’s instructions. The MISSION Lenti miR-590 mimic (HLMIR0815) was purchased from Sigma–Aldrich, and were packaged for transduction to create stable cell lines according to manufacturer’s instructions.

### Quantitative Real-Time PCR

Total isolated RNA from cell cultures was reverse transcribed to complementary cDNAs using Superscript II according to manufacturer’s instructions (Bio-Rad, Hercules, CA, United States). SYBR Green dye-based detection method was used by using the SYBR Green PCR Master Mix assay (Applied Biosystems, Waltham, MA, United States). A series of dilutions of control cDNA were used to generate the standard curves and validate the melting curves for each primer set. Triplicated PCR reactions were carried out for each sample. *GAPDH* was used as a housekeeping gene for normalization. Primers used in the current study were: *VEGF* forward 5′-GAG ATG AGC TTC CTA CAG CAC-3′, reverse 5′-TCA CCG CCT CGG CTT GTC ACA-3′; *GAPDH* forward 5′-AGG GCT GCT TTT AAC TCT GGT-3′, reverse 5′-CCC CAC TTG ATT TTG GAG GGA-3′.

### Dual Luciferase Assay

For the evaluation of miR-590 targeting potential, NSCs were transfected with pGL3 luciferase reporter constructs harboring the miR-590 target sequence (wild type or mutant) for the 3′-UTR of VEGF mRNA. For transcription reporter assay of miR-590, the putative promoter region was selected based on the upstream sequence of miR-590 gene. The promoter region of miR-590 contains one potential HRE, and has been cloned into pGL3 luciferase reporter plasmid at the upstream of Luc open reading frame. Mutagenesis was done by using a QuikChange site-directed mutagenesis kit (Stratagene), and confirmed by DNA sequencing. All transfections were performed by Nucleofector system (Lonza, Basel, Switzerland), and the activities of firefly luciferase and renilla luciferase (as internal control) in the cell lysates were measured with the Dual-Luciferase Assay System (Promega, Madison, WI, United States).

### Western Blot

Western blot was performed in cultured cells following various treatments. The protein lysates (1% NP40, 50 mM Tris, 5 mM EDTA, 1% SDS, 1% sodium deoxycholate, 1% Triton X-100, 1 mM PMSF, 10 mg/ml aprotinin, 1 mg/ml leupeptin, and pH = 7.5) were measured by Bradford assay and the same amount of proteins was resolved on SDS-PAGE followed by an electric transfer to a PVDF membrane. The blots were blocked by 5% non-fat milk and incubated with primary antibodies, including HIF-1α (ab51608, 1:1000; Abcam, Cambridge, MA, United States), VEGF (ab51745, 1:2000; Abcam, United States) and GAPDH (ab9484, 1:1000; Abcam, United States). The blots were then incubated by appropriate HRP conjugated secondary antibodies, and signals were visualized by an enhanced ECL-based imaging system.

### Chromatin Immunoprecipitation (ChIP) Assay

Interaction of HIF-1α to the HREs at the promoter of miR-590 gene was examined by ChIP assay. Briefly, cells were cross-linked with 1% formaldehyde, sheared to an average size of 400 bp, and subsequently immunoprecipitated with antibodies against HIF-1α (Abcam, ab2185) or control IgG. Immunoprecipitated DNA was then isolated for ChIP-PCR using primers for the HRE sites in the promoter region of miR-590. A positive control antibody (RNA polymerase II) and a negative control non-immune IgG were used to demonstrate the efficacy of the kit reagents (Epigentek Group Inc, P-2025-48). Binding efficiency (Bound/RPII%) was calculated by using a ratio of amplification efficiency of the ChIP sample over Polymerase RNA II as follows: 2(RPII CT – Sample CT) × 100%.

### Statistical Analysis

All *in vitro* data were generated from at least three independent experiments, and all *in vivo* data were generated from *n* = 12 rats each experimental group. Values were expressed as means ± SD. Two-tailed Student *t*-test or one-way ANOVA with Tukey’s *post hoc* test were used when appropriate to evaluate the data for statistical comparison, and *P* < 0.05 indicates statistically significant differences.

## Results

### Transplant of FA Pre-treated NSCs Improves Recovery of SCI-Operated Rats

**FIGURE 1:**
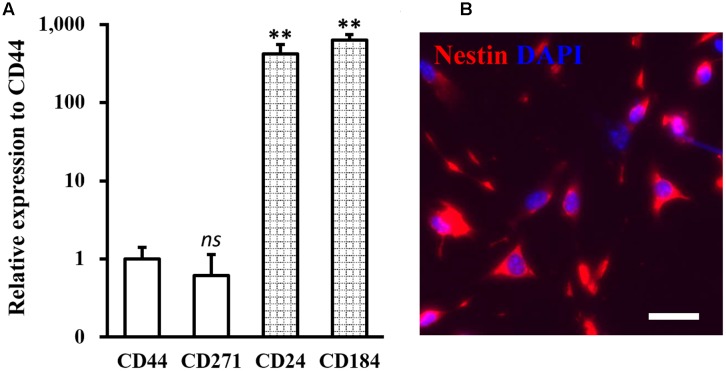
Identity of isolated neural stem cells (NSCs). **(A)** Expressions of NSC specific negative markers CD44 and CD271, and positive markers CD24 and CD184 were analyzed in isolated NSCs. Data were presented as mean ± SD from at least three independent experiments. ^∗∗^*p* < 0.01, *ns* not significant, vs. CD44. **(B)** Immunofluorescence staining of NSC with nestin in red and DAPI in blue. Scale bar 100 μm.

Human NSCs used in the study were isolated from human patients, and expressions of NSC specific markers CD44, CD271, CD24, and CD184 were analyzed. As shown in **Figure 1A**, the isolated NSCs were indeed negative for CD44 and CD271, and positive for CD24 and CD184, confirming their NSC identity (Yuan et al., 2011). Immunofluorescent staining with an NSC-specific marker protein nestin further confirmed the identity of the isolated NSCs (**Figure 1B**).

**FIGURE 2:**
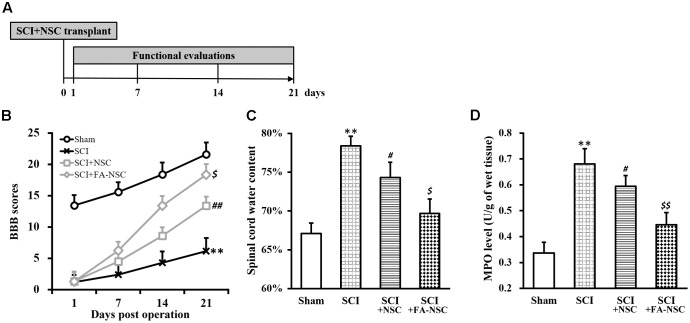
Transplant of ferulic acid (FA) pre-treated NSCs improves recovery of SCI-operated rats. **(A)** Spinal cord injury (SCI) rat model protocol. **(B–D)** Changes in BBB scores **(B)**, spinal cord water content **(C)** and MPO activity **(D)** of SCI rats at indicated days after SCI operation (sham, SCI) and subsequent transplant with NSCs without or with FA pre-treatment (SCI+NSC and SCI+FA-NSC), respectively. Data were presented as mean ± SD from *n* = 12 rats in each experimental group. ^∗∗^*p* < 0.01, vs. sham; ^##^*p* < 0.01, ^#^*p* < 0.05, vs. sham and SCI; ^$$^*p* < 0.01, ^$^*p* < 0.05, vs. SCI and SCI+NSC.

Next, a rat model of SCI was established (**Figure 2A**, and Materials and Methods), and SCI-operated rats received transplant of NSCs with or without FA pre-treatment. We then evaluated the function recovery by monitoring assessing BBB behavioral scores in all four experimental groups of rats. BBB score of SCI-operated rats was significantly reduced throughout the 21 days evaluation period, indicated the successful establishment of SCI rat model (**Figure 2B**). SCI-operated rats also received transplant of untreated NSCs (SCI+NSC) exhibited gradual recovery of BBB scores starting from day 7 to day 21 after SCI operation, compared to that of SCI rats, indicated the effectiveness of NSC transplant therapy in reparing SCI damage. Importantly, SCI rats receiving FA pre-treated NSCs (SCI+FA-NSC) experienced a much more rapid recovery of BBB score. By day 21, BBB score of SCI+FA-NSC rats was significantly higher than SCI+NSC rats, which suggested that FA pre-treatment further enhanced the effect of NSC transplant therapy in reparing SCI.

Water content and myeloperoxidase (MPO) are two indicators of SCI, both of which were reported to significantly increase after SCI (Mbori et al., 2016). Therefore, we also examined levels of these two parameters in the spinal cord (**Figures 2C,D**), and found both parameters exhibited the same trend as that of BBB scores: SCI rats exhibited significantly increased water content and MPO level than sham rats, while SCI+NSC rats displayed recovery to some extent, and SCI+FA-NSC rats showed even more significant recovery compared to SCI+NSC.

### FA Increases VEGF and Decreases miR-590 Expressions in Isolated NSCs

**FIGURE 3:**
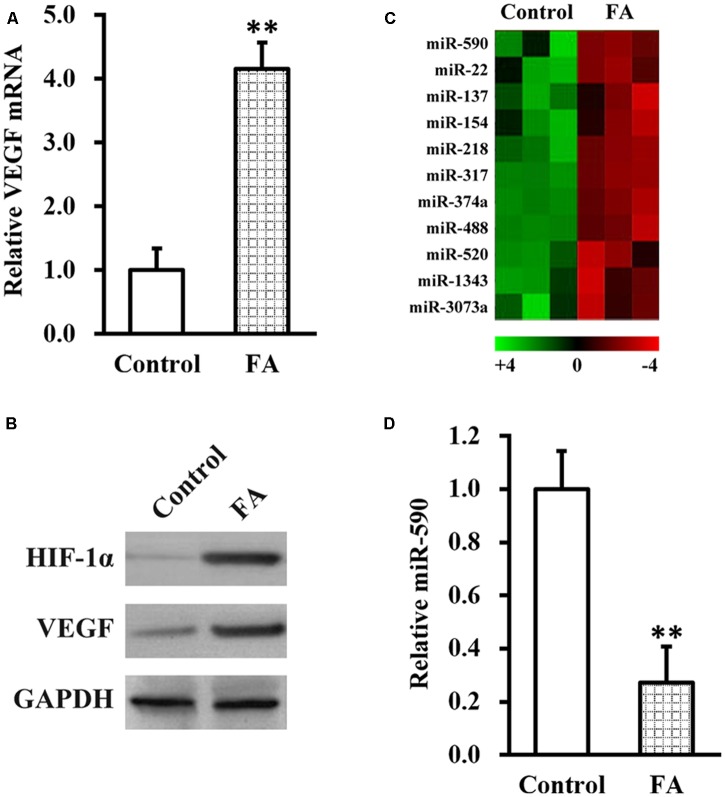
Ferulic acid increases vascular endothelial growth factor (VEGF) and decreases miR-590 expressions in isolated NSCs. Isolated NSCs were treated with either control or 10 μM FA for 24 h, followed by analyses of VEGF mRNA **(A)**, HIF-1α and VEGF protein **(B)** expressions, microRNA microarray **(C)**, and miR-590 expression **(D)**. Data were presented as mean ± SD from at least three independent experiments. ^∗∗^*p* < 0.01, vs. control.

Next, we aimed to uncover the molecular mechanism underlying FA actions in improving SCI recovery following NSC transplant therapy. As a recent study implicated a potential role of hypoxia-induced VEGF in SCI (Long et al., 2015), we therefore examined the effect of FA treatment on expression of VEGF and HIF-1α in the isolated NSC culture. As shown in **Figures 3A,B**, expressions of VEGF mRNA and protein, as well as HIF-1α protein, were significantly increased by FA treatment. We further reasoned that, since microRNA (miRNA, miR) functions to inhibit target gene expression (Bartel, 2009), they might also be involved in FA action and regulate VEGF expression. We therefore performed miRNA microarray analysis in isolated NSCs in the presence or absence of FA treatment, and have found 11 miRNAs downregulated by FA treatment (**Figure 3C**). Among these downregualted miRNAs, miR-590 was particularly interesting because its expression was reported to be negative correlated with VEGF (Zhou et al., 2016). We next verified the microarray data on miR-590 expression with miRNA assay, and confirmed that FA treatment indeed could significantly downregulate miR-590 expression in isolated NSCs (**Figure 3D**).

### MiR-590 Directly Inhibits VEGF Expression by Targeting Its mRNA 3′-UTR

**FIGURE 4:**
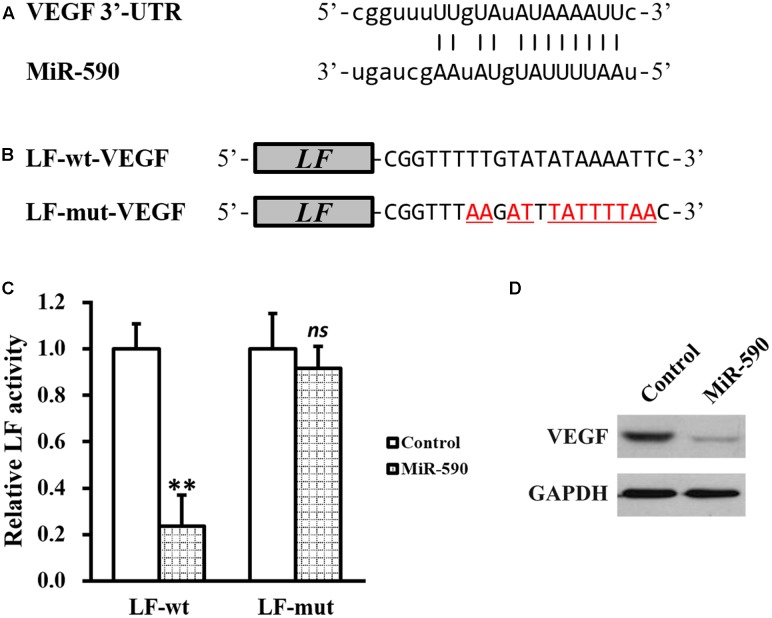
MiR-590 directly inhibits VEGF expression by targeting its mRNA 3′-UTR. **(A)** Sequences of the predicted miR-590 targeting sites on the 3′-UTR of VEGF mRNA. **(B)** Wild type (LF-wt-VEGF) or mutated (LF-mut-VEGF) sequences from VEGF mRNA 3′-UTR were cloned to the 3′-prime of luciferase reporter gene (LF). **(C)** Luciferase activities of LF-wt and LF-mut constructs were assessed in NSCs expressing control or miR-590. **(D)** VEGF protein expression was examined in NSCs expressing control or miR-590. Data were presented as mean ± SD from at least three independent experiments. ^∗∗^*p* < 0.01, *ns* not significant, vs. control.

Given our above results that FA treatment could increase VEGF and decrease miR-590, we next investigated the relationship between the two. By using TargetScanHuman *in silicon* analysis (Lewis et al., 2005), we found a putative miR-590 targeting site on the 3′-UTR of VEGF mRNA (**Figure 4A**). Luciferase reporter assay was then employed to test whether this predicted sequence was responsible for the negative correlation between miR-590 and VEGF, where wild type (LF-wt-VEGF) or mutated (LF-mut-VEGF) sequences from VEGF mRNA 3′-UTR were cloned to the 3′-prime of luciferase reporter gene (**Figure 4B**). Stable expression of miR-590 was also introducted into the isolated NSCs (Supplementary Figure S1). As shown in **Figure 4C**, luciferase activity of LF-wt-VEGF was significantly reduced by miR-590 expression in NSCs, whereas that of LF-mut-VEGF was unaffected, indicating that miR-590 was able to recognize the predicted targeting site on 3′-UTR of VEGF mRNA. Direct examination by Western blot analysis ialso confirmed that VEGF protein was indeed greatly inhibited by miR-590 expression in isolated NSCs (**Figure 4D**).

### Hypoxia Increases VEGF Expression in NSCs via Repressing miR-590

**FIGURE 5:**
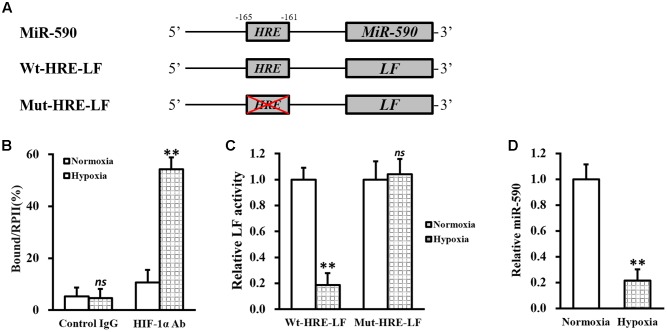
MiR-590 expression in NSCs is repressed by hypoxia via HRE in its promoter region. **(A)** Sequences of the predicted HRE in the promoter region of miR-590 (Wt-HRE-LF) and its mutated version (Mut-HRE-LF) were cloned to the 5′-prime of luciferase reporter gene (LF). **(B)** Isolated NSCs were treated with either 0 (normoxia) or 100 μM CoCl_2_ (hypoxia), respectively, followed by ChIP assay using control IgG or HIF-1α antibody (Ab). **(C)** Luciferase activities of Wt-HRE-LF or Mut-HRE-LF constructs described in **(A)** were assessed in NSCs under normoxia or hypoxia conditions, respectively. **(D)** Expressions of miR-590 was analyzed in NSCs under normoxia or hypoxia conditions, respectively. Data were presented as mean ± SD from at least three independent experiments. ^∗∗^*p* < 0.01, *ns* not significant, vs. control IgG or normoxia.

Besides between miR-590 and VEGF, another negative correlation also existed between miR-590 and hypoxia/HIF-1α. Indeed we found a putative consensus HRE site (Wenger and Gassmann, 1997) in the promoter region of miR-590 gene (**Figure 5A**). Subsequently, a ChIP assay was performed to test the direct binding of HIF-1α to the HRE in miR-590 promoter (**Figure 5B**). Under normoxia condition, a slightly enhanced binding of HIF-1α, compared to control IgG, to the HRE region could be observed. While under hypoxia condition, the binding between HIF-1α and the HRE was markedly increased compared to normoxia. To verify whether expression of miR-590 itself was regulated by hypoxia via this HRE, the predicted wild type HRE sequence (Wt-HRE-LF) and its mutated version (Mut-HRE-LF) were cloned to the 5′-prime of luciferase reporter gene (**Figure 5A**). As shown in **Figure 5C**, luciferase activity of Wt-HRE-LF was significantly reduced by hypoxia in NSCs, whereas Mut-HRE-LF was unchanged. MiRNA assay also confirmed that miR-590 expression was indeed significantly repressed hypoxia (**Figure 5D**). The above results indicated that in isolated NSCs, hypoxia could repress miR-590 via the HRE in its promoter region.

**FIGURE 6:**
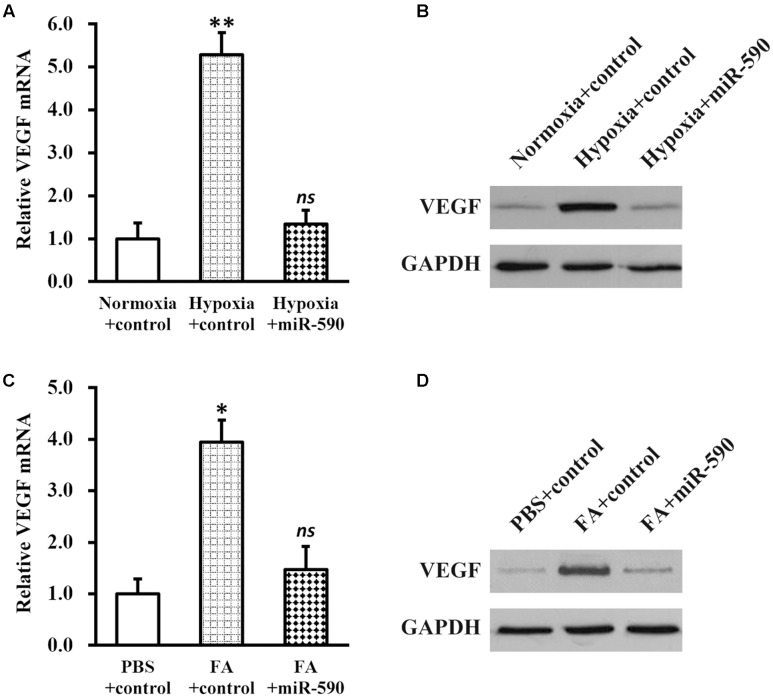
Vascular endothelial growth factor expression in NSCs is elevated via hypoxia-repressed miR-590. **(A,B)** Expressions of VEGF mRNA **(A)** and protein **(B)** were analyzed in NSCs expressing control or miR-590, under normoxia or hypoxia conditions, respectively. Data were presented as mean ± SD from at least three independent experiments. ^∗∗^*p* < 0.01, vs. normoxia+control and hypoxia+miR-590; *ns* not significant, vs. normoxia+control. **(C,D)** Expressions of VEGF mRNA **(C)** and protein **(D)** were analyzed in PBS or FA-treated NSCs expressing control or miR-590, respectively. Data were presented as mean ± SD from at least three independent experiments. ^∗^*p* < 0.05, vs. PBS+control and FA+miR-590; *ns* not significant, vs. PBS+control.

Next, we induced hypoxia in NSCs stably expressing miR-590, and examined the effect on VEGF expression (**Figures 6A,B**). Compared with NSCs expressing control miR under the same conditions, hypoxia failed to elevate either miRNA or protein expressions of VEGF in NSCs expressing miR-590, indicating that hypoxia regulation on VEGF was indirect and was mediated by miR-590 repression. In addition, expression of miR-590 was also able to inhibit FA-induced VEGF upregulation (**Figures 6C,D**), further confirming that miR-590 was required for the effect of FA in inducing VEGF.

### MiR-590 Expression Compromises Recovery of SCI-Operated Rats Following Transplant of FA Pre-treated NSCs

**FIGURE 7:**
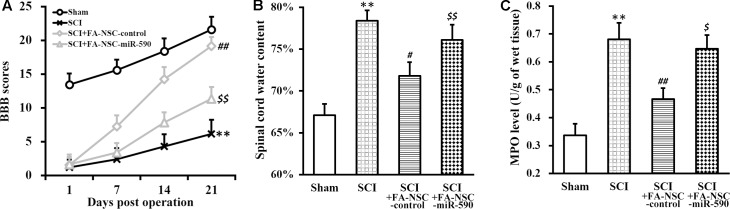
MiR-590 expression compromises recovery of SCI-operated rats following transplant of FA pre-treated NSCs. **(A–C)** Changes in BBB scores **(A)**, spinal cord water content **(B),** and MPO activity **(C)** of SCI rats at indicated days after SCI operation (sham, SCI) and subsequent transplant with FA pre-treated NSCs expressing control or miR-590 (SCI+FA-NSC-control, SCI+FA-NSC-miR-590), respectively. Data were presented as mean ± SD from *n* = 12 rats in each experimental group. ^∗∗^*p* < 0.01, vs. sham; ^##^*p* < 0.01, ^#^*p* < 0.05, vs. sham and SCI; ^$$^*p* < 0.01, ^$^*p* < 0.05, vs. sham and SCI+FA-NSC-control.

To further test whether the above discovered mechanism was responsible for the FA action in improving NSC transplanted therapy, FA pre-treated NSCs expressing either control or miR-590 were transplanted into SCI rats (**Figure 7**). As expected, BBB scores of SCI-FA-NSC-control rats was significantly improved compared to SCI rats receiving no transplant (**Figure 7A**). However, BBB scores of SCI-FA-NSC-miR-590 rats was significantly lower than the SCI-FA-NSC-control group throughout the 21 days evaluation period. Similarly, spinal cord water content and MPO level also exhibited the same trend as BBB scores (**Figures 7B,C**). At the end of day 21, histological analysis of the spinal cord at the injury sites also indicated similar comparisons among the four experimental groups of rats (Supplementary Figure S2). Altogether, these results clearly indicated that miR-590 expression comproised the FA-enhanced recovery process after the transplant.

## Discussion

**FIGURE 8:**
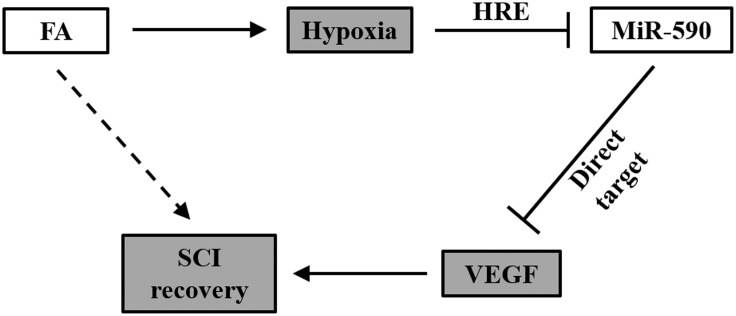
Working model of FA action on SCI recovery. In NSCs, FA induces hypoxia, and subsequently represses miR-590 expression via the HRE in the promoter region, which eventually elevates expression of VEGF that otherwise is inhibited by miR-590. Therefore by increasing VEGF expression, FA is then able to improve the efficacy of NSC transplant therapy to repair SCI.

Summarized in **Figure 8**, data in our present study supports the working model that in isolated human NSCs, FA induces hypoxia, and subsequently represses miR-590 expression via the HRE in the promoter region, which eventually elevates expression of VEGF that otherwise is inhibited by miR-590. Therefore by increasing VEGF expression, FA is then able to improve the efficacy of NSC transplant therapy to repair SCI in a rat model.

MiRNAs are a class of 21–23 nucleotides long, small non-coding RNA molecules, and function as regulatory molecules in many biological processes, mainly by specifically binding to the complementary sequences at 3′-UTR of their target genes (Betel et al., 2010). The main functions of microRNAs include the inhibition of target gene through suppressing mRNA stability and/or translation efficiency, which subsequently may affect many biological processes by negatively modulating gene expressions that share conserved mechanisms (Ameres and Zamore, 2013; Ha and Kim, 2014). In the present study, we speculated that miRNAs could serve as potential signaling relay mechanism between hypoxia and VEGF expression, both of which were induced by FA treatment in isolated NSCs. Indeed, based on the results, miR-590 ideally fits this signaling relay role: (1) miR-590 directly targets 3′-UTR of VEGF mRNA to inhibit VEGF expression; (2) miR-590 expression is negatively regulated by an HRE in its promoter region. The cumulative effect of the above process translates as follows: FA induces hypoxia in isolated NSCs, which then elevates expression of VEGF that otherwise is inhibited by miR-590. Our data not only provide a plausible explanation for FA-induced neurogenesis following NSC transplant in SCI rats, but also implicate miRNA in affecting the NSC transplant therapy that is regulated by FA and/or hypoxia.

Ferulic acid, which exists as natural extract from plants, has been studied and used as a potential therapeutic agent. Particularly in disease models of the neural system, therapies employing FA have been reported to be used for neurodegenerative diseases (Sultana, 2012). For instance, a recent study has supported the role of FA as a promising therapeutic drug in treatment for Alzheimer’s disease, where FA was shown to block amyloid aggregation (Sgarbossa et al., 2015). Importantly, hypoxia induction has been widely reported as the major immediate intracellular effect of FA action both *in vitro* and *in vivo* (Lin et al., 2010; Park and Gerecht, 2014). In line with the above reports, our current study provides yet another instance of FA-induced hypoxia in isolated human NSCs, whose effect persists even after the transplant of the FA pre-treated cells, further suggesting that hypoxia induction by FA could be a conserved signaling pathway across different disease models. However, the exact mode of action that links FA and hypoxia remains unknown, further studies are needed to elucidate this mechanism. Compared with FA, chemicals such as CoCl_2_ that is also able to induce hypoxia are less suitable for animal experiments and clinical applications due to safety concerns. In particular, CoCl_2_ is a prevalent allergen (Zug et al., 2009), therefore its application may cause unexpected adverse effects in animal experiments, let alone in clinical settings. In this context, a safe and natural plant extract, such as FA, would be preferable and hold better potential for future clinical use.

Another factor that has been consistently reported to be associated with both FA and hypoxia is VEGF (Lin et al., 2010; Long et al., 2015). In fact, the role of VEGF in SCI recovery is controversial. On one hand, VEGF contributes to disruption of the blood-spinal cord barrier following SCI. On the other hand, VEGF also promotes angiogenesis, and neurogenesis, both of which are essential for neurologic recovery after SCI. Therefore the temporal and spacial regulations of VEGF expression need to be carefully orchestrated to direct its function toward repairing mechanisms in molecular events that happen following SCI. In this context, our study proposes FA as such an agent, that induces VEGF expression in isolated NSCs and improves the functional recovery following NSC transplant in a rat model of SCI, mediated by hypoxia. Indeed, in our pilot experiments, the dose and duration of FA were found to be important to exert proliferative effect on isolated NSC culture, where 10 μM FA for 24 h exhibited optimal effect (data not shown).

## Author Contributions

Conceived and designed experiments: WL and HY. Provided reagents: WL. Performed experiments: ZL and SW. Analyzed the data: WL. Wrote the paper: HY.

## Conflict of Interest Statement

The authors declare that the research was conducted in the absence of any commercial or financial relationships that could be construed as a potential conflict of interest.
