# Supplementary material for: Ferulic Acid Improves Functional Recovery after Acute Spinal Cord Injury in Rats by Inducing Hypoxia to Inhibit microRNA-590 and Elevate Vascular Endothelial Growth Factor Expressions
Source: Front Mol Neurosci. 2017 Jun 8;10:183. doi: 10.3389/fnmol.2017.00183 (PMC5462975; doi:10.3389/fnmol.2017.00183)
Supplement: Supplementary file 1 [file Image_1.PDF]

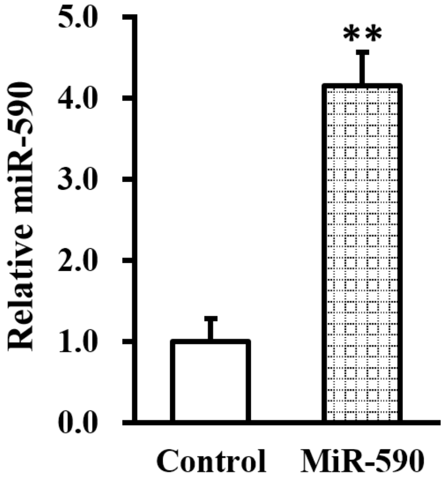

Figure S1. Stable miR-590 expression in isolated NSCs.

Expressions of miR-590 were analyzed by mature miRNA assay in isolated NSCs stably expressing either control or miR-590. Data were presented as mean±SD from at least three independent experiments. ** p<0.01, vs control.

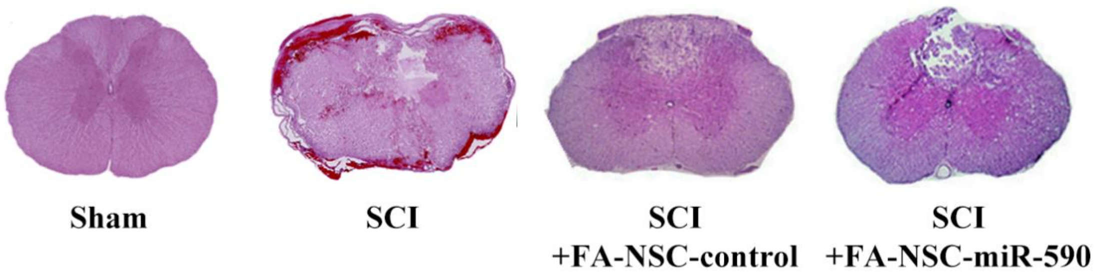

Figure S2. Representative images of histological analysis of spinal cord at the injury sites from rats treated as Figure 7 at the end of day 21.
